# 10-TaGA: A Triaging Tool for Geriatric-Informed Heart Failure Care

**DOI:** 10.1016/j.jacadv.2025.102126

**Published:** 2025-09-17

**Authors:** Eiran Z. Gorodeski, Vincent D. Salvador

**Affiliations:** University Hospitals Harrington Heart & Vascular Institute, Case Western Reserve University School of Medicine, Cleveland, Ohio, USA

**Keywords:** geriatric assessment, geriatric syndromes, heart failure, risk prediction

**Figure:**
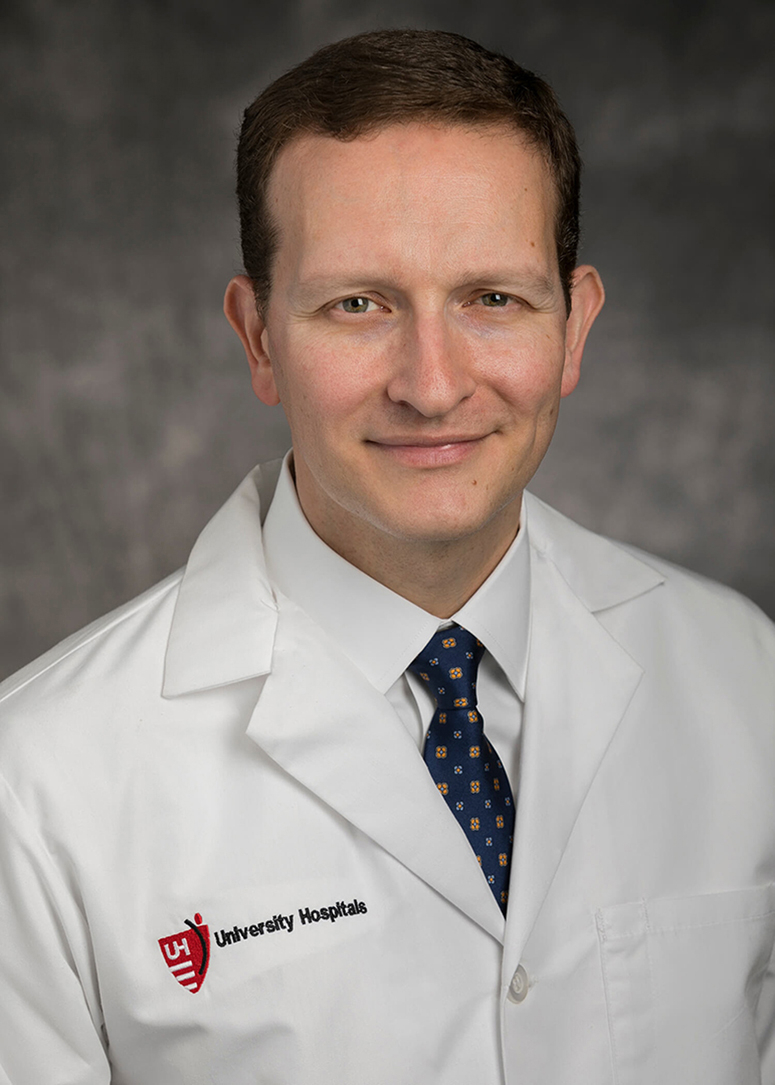

The rapidly growing older adult population brings with it a rising burden of heart failure (HF), increased health care utilization, and complex medical needs that challenge health care systems.^1^^,^^2^ Traditional health assessments have been cardiovascular disease-centric, focusing primarily on major cardiovascular events and mortality as outcomes. However, older adults are a heterogeneous patient population with distinct needs and priorities that go beyond just adverse cardiac events and mortality. Often excluded from major cardiovascular clinical trials, older adults require patient-centered outcomes that include preserving physical function and cognitive health, maintaining independence, and enhancing quality of life as they age. These outcomes are as important as—if not more than—traditional clinical cardiovascular endpoints.

Recognizing the limitations of relying solely on chronological age in treatment decisions, comprehensive geriatric assessment (CGA) provides a multidomain method for evaluating the health of older adults.^3^ This method aligns with the geriatric 4Ms framework: mind, medications, mobility, and what matters most, especially in the context of multimorbidity.^4^

However, CGA requires dedicated time and resources that are often unavailable in clinical settings. Ideally, it involves using both self-report and performance-based tests with caregiver input, but practical challenges exist. Several strategies have been suggested, including conducting focused assessments during office visits or administering quick screenings followed by comprehensive evaluations when needed.^5^ This creates an inherent tension between brevity and comprehensiveness. Since routine CGA in outpatient settings is often impractical, time-consuming, and not consistently correlated with improved patient outcomes,^6^ there is a clear need for more efficient alternative approaches.

In their latest study published in the *Journal*, Tavares et al evaluated 10-TaGA as a targeted geriatric screening tool for older adults with HF, excluding those with acute events requiring direct hospital admission.^7^ Unlike traditional HF-specific risk models such as the Meta-Analysis Global Group in Chronic Heart Failure (MAGGIC) risk score and the Seattle Heart Failure Model, 10-TaGA notably does not include age as a variable.

Initially developed through expert consensus of geriatricians in Brazil, 10-TaGA is a concise multidimensional evaluation tool based on several key domains, including cognitive and emotional status, functional impairment, psychosocial factors, nutritional status, and polypharmacy burden. The tool combines self-reported measures and performance-based tests to generate a single numerical score (from 0 to 1) that reflects overall impairment in older adults (Figure 1). Higher scores indicate greater cumulative deficits in health status.^8^

**Figure 1:**
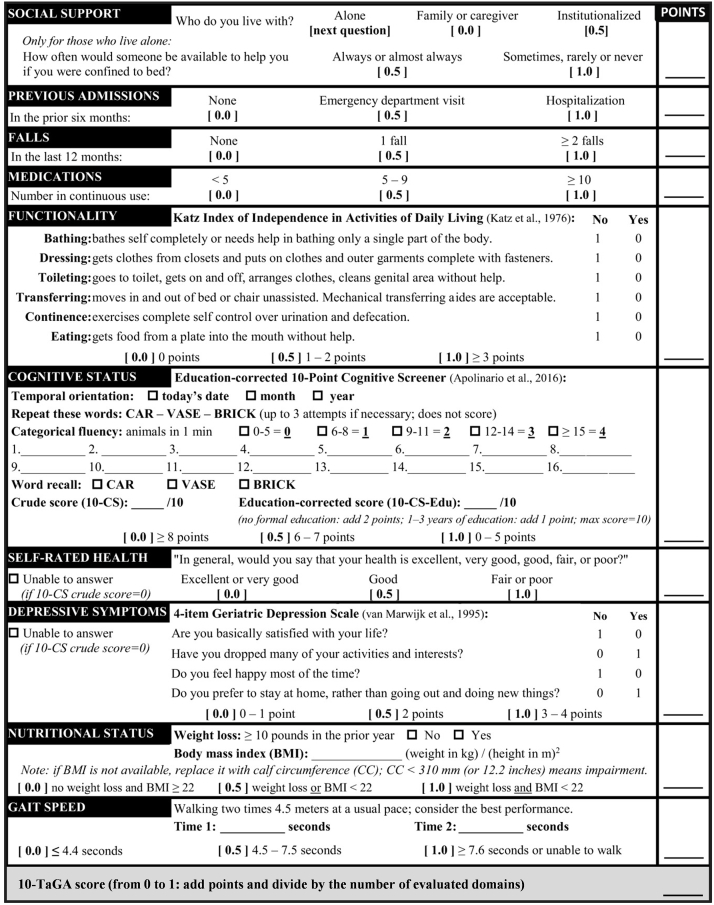
Instructions for Administration and Scoring of the 10-Domain Targeted Geriatric Assessment Tool (10-TaGA) Adapted from Aliberti MJR, et al. J Am Geriatr Soc. 2018 with permission.^8^

The researchers followed a prospective cohort of 326 adults (mean age: 80.9 ± 7.7 years) with various phenotypes of HF admitted to acute short-term care in São Paulo, Brazil. They tracked the primary outcome of composite all-cause mortality and unplanned hospitalization over 1 year. Notably, they also reported “days of health lost” due to health care utilization and death—a metric particularly relevant to older adults. The cohort included older adults deemed high risk for adverse outcomes, as shown by severe impairment in gait speed and significant burden of polypharmacy. When categorized by tertiles of 10-TaGA score, approximately 24% were classified as high risk, 36% as intermediate risk, and 40% as low risk for adverse clinical outcomes.^7^

The results showed a statistically significant two-fold increase in all-cause mortality and hospitalization in the high-risk group compared to the low-risk group, with clear gradation of risk based on 10-TaGA scores. Additionally, combining 10-TaGA with other clinical and HF-related risk scores enhanced the discriminatory performance of their model. A similar pattern was seen with increased lost days in high-risk groups. Interestingly, there was only a weak correlation between 10-TaGA scores and HF-specific risk scores and NYHA functional class, indicating that 10-TaGA captures different aspects of patient vulnerability.

The study demonstrates the practicality of using 10-TaGA as a quick, easy-to-administer screening tool for geriatric conditions, especially in busy clinical settings. By using 10-TaGA for prognostication in HF, the research highlights the importance of geriatric assessment in managing HF that coexists with other comorbidities in older adults. Since these patients are at high risk for hospital readmissions due to multiple conditions, screening older adults with HF for other geriatric syndromes could be incorporated into strategies to reduce readmissions during postdischarge follow-ups.

However, caution must be exercised when interpreting the numerical score. The 10-TaGA score is not meant to be a fixed, static risk score of overall HF disease trajectory, which is inherently dynamic. Instead, it requires careful interpretation by examining the details of the screening evaluation and identifying deficits in various domains that warrant intervention through a multidisciplinary approach. The true value of 10-TaGA extends beyond simple prediction of adverse outcomes. It acts as a targeted screening tool designed to guide more detailed assessment and management of geriatric syndromes such as frailty, functional impairment, cognitive decline, fall risk, malnutrition, depression, social isolation, and polypharmacy.

The primary purpose of conducting geriatric assessment is both risk stratification and guiding treatment decisions to improve patient-centered outcomes. Performing a geriatric assessment should ideally support discussions about overall health status within a shared decision-making framework—an approach that has been successfully promoted in other fields such as oncology.^9^

Using targeted geriatric assessment represents only half the challenge in managing chronic disease like HF. Increased awareness of other competing medical and nonmedical issues in older adults with HF must be translated into providing individualized treatment plans. It is crucial to include discussions of “what matters most” after identifying geriatric syndromes. This helps align targeted interventions based on patients' goals, values, and preferences—a core principle of geriatric care.^4^

The discussion should help identify personalized health outcome goals and treatment options within a shared decision-making framework. Input from other specialty services will be vital in providing a comprehensive treatment approach alongside HF guideline-directed medical therapy.

Another key point is the need to evaluate decisional capacity regarding expected prognosis and appropriate treatment as disease progresses, which can significantly impair cognitive functioning over time.^10^

Several important limitations must be considered when interpreting the study results. First, this single-center study had a relatively small sample size and a limited number of total events for all-cause mortality and unplanned hospitalization. Further validation of its predictive performance across diverse older adult populations is needed before widespread implementation. Second, external validity is limited. It remains uncertain whether these findings can be generalized to inpatient, emergency department, or skilled nursing facility settings. Third, the tool itself has modest discriminatory performance. While there was statistically significant improvement in discrimination for hard outcomes when 10-TaGA was added to other clinical variables and HF-specific risk models, the overall discriminatory ability remains in the fair range for clinical usefulness. Fourth, temporal considerations must be taken to account. The 10-TaGA score offers just a snapshot at one specific point in time regarding the overall risk of adverse outcomes in HF. Because HF disease trajectory is variable for older adults,^11^ the overall risk status and vulnerability are likely to change as other geriatric conditions develop. Relying solely on a numerical risk score could lead to a false impression of low risk. Finally, there is uncertainty about how much each domain-specific variable contributes to the overall risk. Which geriatric domains have greater impact on outcomes in specific HF disease courses remains unclear and warrants further investigation in future studies.

The aging population demands an evolving cardiovascular approach that is responsive to its unique needs. Incorporating geriatric assessment should become a core component of HF care, not an optional addition. It should be considered a vital part of determining prognosis and treatment pathways for older adults with HF. A geriatric assessment-guided HF care pathway may soon become standard as demographics shift, emphasizing patient-centered outcomes and increasing demands on health care systems to deliver high-value care.^12^

Now more than ever, advancing knowledge on implementing geriatric assessment in HF management through a team-based multidisciplinary approach warrants further exploration. There is a pressing need to develop scalable models that can be easily integrated into routine cardiology practice. Digital health technology combined with artificial intelligence—specifically the use of “gerotechnology” as an adjunct to clinical assessment—presents another promising opportunity that could yield valuable data for designing individualized treatment plans.^13^

10-TaGA holds potential usefulness not only as the next HF prognostic indicator but, more importantly, as an HF evaluation checklist serving as a guide to more in-depth assessment of geriatric domains of concern. Far from being redundant with other traditional HF risk models, it offers unique data critical to designing multimodal, patient-centered interventions beyond cardiac-specific therapies. After all, the actual value of any prognostic variable lies in how it influences the management plan by identifying vulnerabilities associated with aging.

A targeted geriatric assessment is not a substitute for traditional CGA when indicated and requires nuanced interpretation of focused geriatric assessment findings. Relevant information gathered from the evaluation should be used to facilitate optimization of patient-centered outcomes by utilizing multidisciplinary team expertise and involving family members or caregivers.

This structured approach requires thoughtful implementation to integrate into clinical workflow, supported by enabling system policies, reimbursement models, and promotion of fundamental geriatric competencies among HF clinicians. Only through such comprehensive integration can we hope to address the complex needs of our aging population with HF.

## Funding support and author disclosures

The authors have reported that they have no relationships relevant to the contents of this paper to disclose.

## References

[1] Heidenreich P.A., Bozkurt B., Aguilar D. (2022). 2022 AHA/ACC/HFSA guideline for the management of heart failure: a report of the American college of cardiology/American heart association joint committee on clinical practice guidelines. J Am Coll Cardiol.

[2] Vespa J.U.C. The U.S. joins other countries with large aging populations. Census.gov. https://www.census.gov/library/stories/2018/03/graying-america.html.

[3] Halter J., Ouslander J., Studenski S. (2022). Hazzard’s Geriatric Medicine and Gerontology.

[4] Institute for Healthcare Improvement (2022). Age-Friendly Health Systems: Guide to Using the 4Ms in the Care of Older Adults.

[5] Tatum I.P.E., Talebreza S., Ross J.S. (2018). Geriatric assessment: an office-based approach. Am Fam Physician.

[6] Briggs R., McDonough A., Ellis G., Bennett K., O’Neill D., Robinson D. (2022). Comprehensive geriatric assessment for community-dwelling, high-risk, frail, older people. Cochrane Database Syst Rev.

[7] Tavares C.A.M., Meyer J.O.F., Hummel S.L. (2025). Targeted geriatric assessment to predict outcomes in older adults with heart failure. JACC Adv.

[8] Aliberti M.J.R., Apolinario D., Suemoto C.K. (2018). Targeted geriatric assessment for fast-paced healthcare settings: development, validity, and reliability. J Am Geriatr Soc.

[9] Dale W., Klepin H.D., Williams G.R. (2023). Practical assessment and management of vulnerabilities in older patients receiving systemic cancer therapy: ASCO guideline update. J Clin Oncol.

[10] Goyal P., Didomenico R.J., Pressler S.J. (2024). Cognitive impairment in heart failure: a heart failure society of America scientific statement. J Card Fail.

[11] Goodlin S.J. (2009). Palliative care in congestive heart failure. J Am Coll Cardiol.

[12] Gorodeski E.Z., Goyal P., Hummel S.L. (2018). Domain management approach to heart failure in the geriatric patient: present and future. J Am Coll Cardiol.

[13] Krishnaswami A., Beavers C., Dorsch M.P. (2020). Gerotechnology for older adults with cardiovascular diseases: JACC state-of-the-art review. J Am Coll Cardiol.

